# Sandwich Immunoassays of Multicomponent Subtrace Pathogenic DNA Based on Magnetic Fluorescent Encoded Nanoparticles

**DOI:** 10.1155/2016/7324384

**Published:** 2016-01-11

**Authors:** Yaxu Liu, Xuanjun Zhang, Yifeng E, Fang Fang, Guangkai Kuang, Guannan Wang

**Affiliations:** ^1^Department of Chemistry, College of Pharmacy, Liaoning Medical University, Jinzhou 121001, China; ^2^Faculty of Health Sciences, University of Macau, Avenida da Universidade, Taipa, Macau

## Abstract

A novel magnetic fluorescent encoded nanoimmunoassay system for multicomponent detection and separation of the subtrace pathogenic DNA (hepatitis B virus surface gene, HBV; hepatitis A virus poly the protein gene, HAV) was established based on new type of magnetic fluorescent encoded nanoparticles and sandwich immunoassay principle. This method combines multifunctional nanoparticles, immunoassay technique, fluorescence labeling, and magnetic separation of multicomponent technology. It has many advantages such as high sensitivity, low detection limit, easy operation, and great potential for development. The results of this work show that, based on nanoimmunoassay system, it could quantitatively detect the multicomponent trace pathogenic HAV and HBV DNA, as well as detection limit up to 0.1 pM and 0.12 pM. Furthermore, with the improvement of the performances of magnetic fluorescent encoded nanoparticles, the sensitivity will be further improved. In this experiment, a new nanoimmunoassay system based on magnetic fluorescent encoded nanoparticles was established, which will provide a new way for the immunoassay and separation of multicomponent biomolecules.

## 1. Introduction

In recent years, multivariate analysis and separation technology, analysis and separation of multicomponent biological molecular in a single sample, has been a major research for detection of gene expression in genetic and infectious disease [1], drug recognition [2], environmental monitoring [3], and food safety detection [4]. With the development of nanomaterials and nanotechnology, the research and application of multifunctional nanomaterials have attracted considerable attention in multivariate analysis [5–7]. Superparamagnetic nanoparticles, especially Fe_3_O_4_ nanoparticles, have played a pivotal role among MRI, biomagnetic separation, targeted drug delivery, magnetic hyperthermia, and immobilized enzyme due to their excellent physical properties and biological applications [8–11]. Quantum dots (QDs) have been widely applied to the research of fluorescent hybridization analysis, cell imaging, and living tracking as an outstanding fluorescent indicator due to their superior properties, such as special optical property, high photostability, and size-tunable light emission [12–16].

Magnetic fluorescent encoded nanoparticles are a kind of novel multifunctional nanoparticle with magnetic and fluorescent encoding properties, being the attractive nanomaterial in multivariate analysis and separation technology due to its excellent characteristics, such as the integration of the advantages of magnetism and fluorescent encoding, multicomponent labeling, and targeted separation [17–20]. Recently, the design, synthesis, functionalization, and application of magnetic fluorescent encoded nanoparticles have attracted much attention. However, the fabrication of this system is complex and rarely reported due to the interaction of various nanoparticles in one final nanoparticle [21–24]; particularly, as far as we know, there are very few reports about the combination of magnetic fluorescent encoded nanoparticles and sandwich immunoassay.

In this work, we prepared magnetic fluorescent encoded nanoparticles based on superparamagnetic Fe_3_O_4_ nanoparticles and two quantum dots with different emission wavelength by reverse microemulsion method. And then, magnetic fluorescent encoded nanoimmunoassay system was established by combination of the nanoparticles and sandwich immunoassay for multicomponent biological assay and separation. In this work, the subtrace hepatitis B virus surface antigen gene (HBV) and hepatitis A virus Vall7 polyprotein gene (HAV) as pathogenic DNA were successfully detected and separated, respectively. The results show that this new magnetic fluorescent encoded nanoimmunoassay system can be generally used to multicomponent biological immunoassay and separation of other biomolecules based on its high sensitivity, lower cost, easy operation, and time saving.

## 2. Experimental

### 2.1. Materials and Instrumentation

Cyclohexane, Triton X-100, *n*-hexanol, acetone, ethanol, cadmium chloride (CdCl_2_), sodium borohydride (NaBH_4_), iron (III) chloride hexahydrate (FeCl_3_·6H_2_O), and iron (II) chloride tetrahydrate were purchased from Tianjin Chemical Reagents Factory (China). Tellurium (reagent powder), mercaptosuccinic acid, tetramethylammonium hydroxide (TMA, 25%), tetraethoxysilane (TEOS), 3-aminopropyltrimethoxysilane (APS), poly(diallyldimethylammonium chloride) (PDDA, *M*_*w*_ = 70000 g/mol), and 3-(trihydroxysilyl)-propyl methyl-phosphonate (THPMP) were supplied by Sigma-Aldrich Co., Ltd. (United States). All chemicals were used of analytical reagent grade, and the water used in this study was redistilled water.

The targeted DNA was designed from hepatitis B surface antigen gene (HBV) and hepatitis A virus Vall7 polyprotein gene (HAV). The single-stranded DNA (freeze-dried powder) about HBV was provided by TaKaRa Biotechnology Co., Ltd. (Dalian, China). The base sequences of single-stranded DNA were as follows:

 HBV:
   Capture DNA: 3′-AAC CGA AAG TCA ATA-5′.
   Target DNA: 5′-TTG GCT TTC AGT TAT-ATG GAT GAT GTG GTA-3′.
   Complement DNA: 3′-TAC CTA CTA CAC CAT-FITC-5′.
 HAV:
   Capture DNA: 3′-AAT CTC AAC GTA CCT-5′.
   Target DNA: 5′-TTA GAG TTG CAT GGA-TTA ACT CCT CTT TCT-3′.
   Complement DNA: 3′-AAT TGA GGA GAA AGA-FITC-5′.

Additionally, wash buffer (WB, 10 mM Tris-HCl, pH 7, 1 mM EDTA), binding buffer (BB, 10 mM Tri-HCl, pH 7, 1.0 M NaCl, 2 mM EDTA), and PBS buffer solution (PH = 7.4) were fabricated by our laboratory.

The fluorescence spectra were recorded with a fluorescence spectrophotometer (RF-5301, Shimadzu Co., Japan). The UV-vis absorption spectra were measured by a UV-vis spectrometer (GBC Cintra 10e, Varian Co., United States). The composite nanoparticles were dispersed by a bath ultrasonic cleaner (Autoscience AS 3120, Tianjin, China). The microscopic structures were obtained using a transmission electron microscope (TEM) (JEOL-1230, Japan). The magnetic hysteresis loops were performed on a vibrating sample magnetometer (VSM) (Nanjing Nanda Instrument Plant, China). The zeta potential and dynamic light scattering (DLS) size distribution was characterized by a Malvern Zetasizer ZEN 3600. All optical measurements were carried out at room temperature under ambient conditions.

### 2.2. Preparation of Amino-Modified Magnetic Fluorescent Composite Nanoparticles

Stable water compatible CdTe quantum dots (QDs) and superparamagnetic Fe_3_O_4_ nanoparticles were synthesized as described in our previous work [25, 26]. The CdTe QDs with emission maximum at 573 nm were QDs_1_ (average diameter 3.7 nm), and CdTe QDs with emission maximum at 653 nm were QDs_2_ (average diameter 4.3 nm), and the concentration was 2 × 10^−3^ mol/L. Superparamagnetic Fe_3_O_4_ nanoparticles were synthesized by chemical coprecipitation method, and the harvested concentrations, diameter, and saturation magnetization value of nanoparticles were 10 mmol/L, 7–12 nm, and 60 emu/g, respectively.

QDs_1_-Fe_3_O_4_/SiO_2_ composite nanoparticles were synthesized by reverse microemulsion method at room temperature. At first, 7.5 mL cyclohexane, 1.77 mL Triton X-100, 1.8 mL *n*-hexanol, 60 *μ*L PDDA solution (0.075% v/v), and 100 *μ*L TEOS were added in a flask including 100 *μ*L Fe_3_O_4_ nanoparticles and 400 *μ*L CdTe QDs_1_; after stirring for 0.5 h, a uniform microemulsion system was formed. Subsequently, 60 *μ*L NH_4_OH (28% v/v) was added to the microemulsion system to initiate TEOS hydrolysis in the dark at room temperature. After reaction for 24 h, 20 *μ*L APS and 40 *μ*L THPMP were added to the system; the reaction system was kept under stirring for 24 h at room temperature. Next, 20 mL of acetone was added in the flask to break the microemulsion system, and the resultant mixture was amino-modified magnetic fluorescent nanospheres (QDs_1_-Fe_3_O_4_/SiO_2_ composite nanoparticles) which was separated by a magnet and washed three times in sequence with ethanol and water, respectively. Ultimately, QDs_1_-Fe_3_O_4_/SiO_2_ composite nanoparticles were diluted in 5 mL water, and the harvested concentration of the composite nanoparticles was 2.2 mg/mL.

### 2.3. Synthesized Amino Functional Magnetic Fluorescent Encoded Nanoparticles (AFMFEM)

The preparation of AFMFEM was according to our previous work [27]. 7.5 mL cyclohexane, 1.8 mL *n*-hexanol, 1.77 mL Triton X-100, a certain proportion QDs_1_ solution, and the as-synthesized amino-modified QDs_1_-Fe_3_O_4_/SiO_2_ solution were added in a 50 mL flask including 100 *μ*L TEOS; after stirring for 30 min, the microemulsion system was formed. Then, 120 *μ*L NH_3_·H_2_O (28% v/v) was added into the flask, and after stirring for 24 h, 30 *μ*L APS and 60 *μ*L THPMP were added to the system. After stirring for 24 h, 20 mL of acetone was added into the flask to break the microemulsion system, and the stirring was terminated when a great deal of precipitation was formed. A magnet was used to separate the product which was washed successively three times with ethanol and water. Finally, the final product was AFMFEM which was diluted in 5 mL deionized water, and the harvested concentration of AFMFEM was 3.6 mg/mL.

According to the requirement of this work, two types of the AFMFEM, with fluorescent encoded *I*_560 nm_ : *I*_650 nm_ = 2 : 1 and *I*_560 nm_ : *I*_650 nm_ = 4 : 5, were chosen as models of AFMFEM-1 and AFMFEM-2 for following works.

### 2.4. Preparation of Capture DNA-Modified Magnetic Fluorescent Encoded Nanoprobes

The capture DNA-AFMFEM was fabricated by electrostatic adsorption of amino functional magnetic fluorescent encoded nanoparticles and capture DNA. Capture DNA and AFMFEM were combined by the electrostatic adsorption principle because of positive charges on the surface of AFMFEM and negative charges on the framework of single-stranded DNA. First, 100 *μ*L AFMFEM (3.6 mg/mL) was washed with wash buffer (WB, 10 mM Tris-HCl, pH 7, 1 mM EDTA) and distributed in 500 *μ*L WB solution. Then, different volumes of capture DNA (200 nM/L) were added, respectively, to the equal AFMFEM solutions, and the mixture solution was diluted to 1 mL. The mixture solution was shaken for 60 min at room temperature, and the final product was capture DNA-AFMFEM which was separated by a magnet. The capture DNA-AFMFEM were washed repetitively with WB solution and were dispersed in 100 *μ*L PBS solution.

Two types of magnetic fluorescent encoded nanoprobes were synthesized as models of AFMFEM-1-HAV capture DNA and AFMFEM-2-HAV capture DNA via the above method.

### 2.5. Fabrication of Hybrid Compound Using Sandwich Hybridization Analysis

**Scheme 1:**
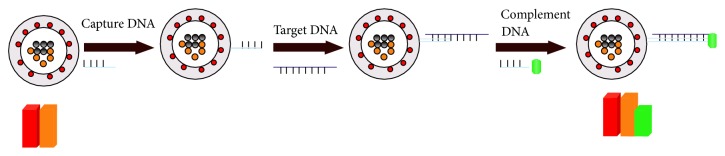
Scheme for sandwich immunoassay determination of DNA based on magnetic fluorescent encoded nanoparticles.

In order to determine unlabeled HAV Target DNA and HBV Target DNA, the sandwich hybridization analysis was used in this work, as schematically outlined in Scheme 1. 50 *μ*L capture DNA-AFMFEM nanoprobes were mixed with equal, various volumes of Target DNA and FITC-Complement DNA, and BB solution was added to the system. The mixture solution was stirred slowly at room temperature to promote the hybridization process of capture DNA and Complement DNA with Target DNA. After stirring for 2 h, the composite was separated by a magnet. The product was washed two times with WB solution and was dispersed in 1 mL PBS buffer solution. Finally, fluorescence image of the product was measured.

Two types of hybrid composite were fabricated as models of AFMFEM-1-HAV capture DNA/HAV Target DNA/HAV FITC-Complement DNA and AFMFEM-2-HBV capture DNA/HBV Target DNA/HBV FITC-Complement DNA via the above method.

## 3. Results and Discussion

### 3.1. Characterization of AFMFEM

**Figure 1:**
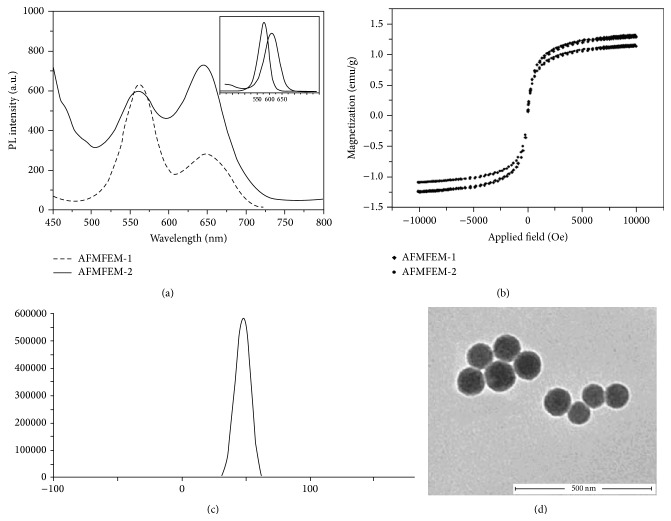
The fluorescence spectra of AFMFEM-1 and AFMFEM-2 (a): the picture in the upper right corner is the fluorescence spectra of QDs_1_ and QDs_2_; the hysteresis loops taken at 300 K for AFMFEM-1 and AFMFEM-2 (b); the zeta potential of AFMFEM (c); the TEM micrographs of AFMFEM (d).

In order to avoid the spectra overlap, in this work, orange QDs_1_ (emission maximum at 573 nm) and red QDs_2_ (emission maximum at 653 nm) were chosen to fabricate magnetic fluorescent encoded nanoparticles.  Figure 1(a) shows the emission spectra obtained from QDs and two types of AFMFEM, and the insert is fluorescence spectra of two types of QDs. It can be seen that two types of AFMFEM have clear fluorescent encoding and high fluorescence intensity owing to using different amount of QDs_1_-Fe_3_O_4_/SiO_2_ and QDs_2_, which are the good candidates as fluorescent probe.  Figure 1(b) shows the hysteresis loop of two types of AFMFEM at room temperature, and it can be seen that two types of AFMFEM have perfect superparamagnetic and magnetic response property, and the saturation magnetization value is, respectively, 1.21 emu/g and 1.44 emu/g, manifesting that the trace pathogenic DNA can be separated by the magnetic field.

The properties of AFMFEM could decide the ability of enrichment, detection, and separation of DNA, and the amounts of amino grafted on the nanoparticle surfaces are especially important, because the combination between capture DNA and AFMFEM was formed through electrostatic binding. The more amounts of the amino on the surface, the more amounts of AFMFEM that could combine with capture DNA, and the stronger ability of enrichment and detection of Target DNA. The amounts of the amino grafted on AFMFEM can be evaluated through zeta potential detection. Figures 1(c) and 1(d) show, respectively, zeta potential spectra and TEM image of AFMFEM. From Figure 1(c), it can be seen that zeta potential of AFMFEM at neutral pH was +46.5 mV, which implied a large quantity of amino on the surface of AFMFEM, and the AFMFEM could disperse well in the water because of electrostatic repulsion. From Figure 1(d), it can be seen that the formed AFMFEM have uniform size about 100 ± 10 nm and good dispersity.

### 3.2. Preparation of Capture DNA-AFMFEM Probe

**Figure 2:**
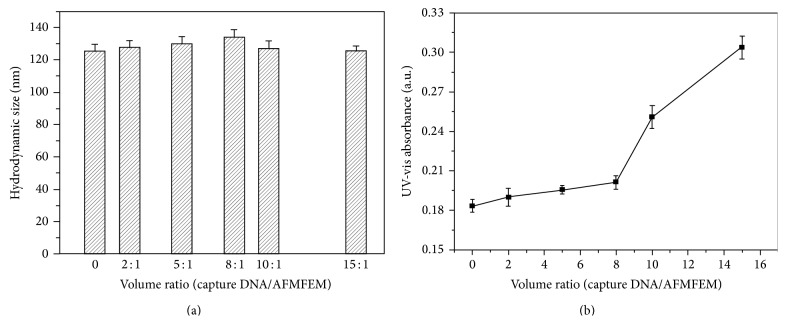
DLS analysis of capture DNA-AFMFEM probe prepared at various volume ratios of capture DNA (200 nM) to 10 *μ*L AFMFEM (3.6 mg/mL) (a); the adsorption spectra at 260 nm of the supernatant of the mixture with various volume ratios (DNA/AFMN) (b).

In order to optimize the combination of capture DNA with AFMFEM, different volumes of capture DNA (200 nM) were reacted with 10 *μ*L AFMFEM solution (3.6 mg/mL); the product was separated and measured by DLS.  Figure 2(a) shows the hydrated radius of capture DNA-AFMFEM in solution at the various proportions of capture DNA and AFMFEM. From Figure 2(a), it can be seen that the hydrated radius of AFMFEM is about 125 ± 5 nm when there is no capture DNA, and the hydrated radius of capture DNA-AFMFEM increases gradually as capture DNA was added abidingly. When the proportion of capture DNA and AFMFEM reaches 8 : 1, we get the maximum hydrated radius in 135 nm. The result seems to be comparatively reasonable if hydrodynamic sizes of AFMFEM (130 nm) and capture DNA (2 nm) are considered. The volume of AFMFEM is slightly increased because the electrostatic force causes the DNA to pack tightly on the surface of AFMFEM. When the volume ratio of capture DNA and AFMFEM reaches 8 : 1, the adsorption of capture DNA on the AFMFEM reaches saturation. The hydrated radius of the product began to decrease due to the presence of excessive free capture DNA as capture DNA was added abidingly.

To further verify the measurement of DLS, the UV adsorption spectra of supernatant of the mixture with various volume ratio after magnetic separation were measured, to monitor the quantity of excessive free capture DNA.  Figure 2(b) shows the results. From Figure 2(b), it can be seen that the UV adsorption of supernatant (260 nm) was almost the same for the volume ratio from 1 : 1 to 8 : 1, which indicates a little of excessive DNA dispersed in the supernatant. When the volume ratio is higher than 8 : 1, the UV absorption of supernatant increased rapidly with the volume ratio, which proves amounts of excessive DNA dispersed in the solution, and this phenomenon further indicates the capture DNA on the surface of AFMFEM reached saturation when the ratio is 8 : 1.

### 3.3. Nonspecific Hybridization of Capture DNA-AFMFEM-1 and Target DNA

**Figure 3:**
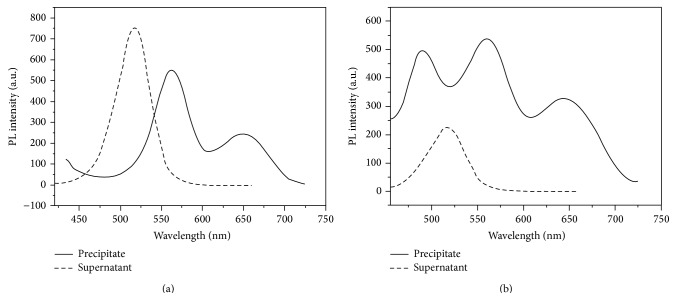
The investigation of nonspecific binding: HAV capture DNA-AFMFEM-1 with HBV Target DNA and HBV FITC-Complement DNA (a); HAV capture DNA-AFMFEM-1 with HAV Target DNA and HAV FITC-Complement DNA (b).

For studying of DNA specific hybridization reaction, two groups of experiments were performed: in the first group, the HBV Target DNA and the HBV FITC-Complement DNA were used to connect with HAV capture DNA-AFMFEM-1; in second group, the HAV Target DNA and the HAV FITC-Complement DNA were used to react with HAV capture DNA AFMFEM-1. The magnet was used to separate products and the fluorescence spectra of precipitation and supernatant were recorded.  Figure 3(a) shows the results of the first group. The supernatant exhibits strong fluorescence peak at 510 nm, but there is no obvious fluorescence peak at 510 nm in the spectra of the precipitation, which proved that the hybridization reaction did not occur between HAV capture DNA AFMFEM-1 and the mixture of HBV Target DNA. But from Figure 3(b), the results of the second group, the spectra of precipitation separated by magnet show a strong fluorescence peak at 510 nm and the supernatant also exhibits strong fluorescence peak at 510 nm but lower than that of Figure 3(a); it proved that the hybridization reaction has indeed occurred between HAV capture DNA AFMFEM-1 and the mixture of HAV Target DNA. The results of Figure 3 clearly demonstrate that HAV Target DNA and HBV Target DNA do not interfere with each other in the same system and guarantee the process of multicomponent immunoassay.

### 3.4. Enrichment and Detection of Subtrace Target DNA

**Figure 4:**
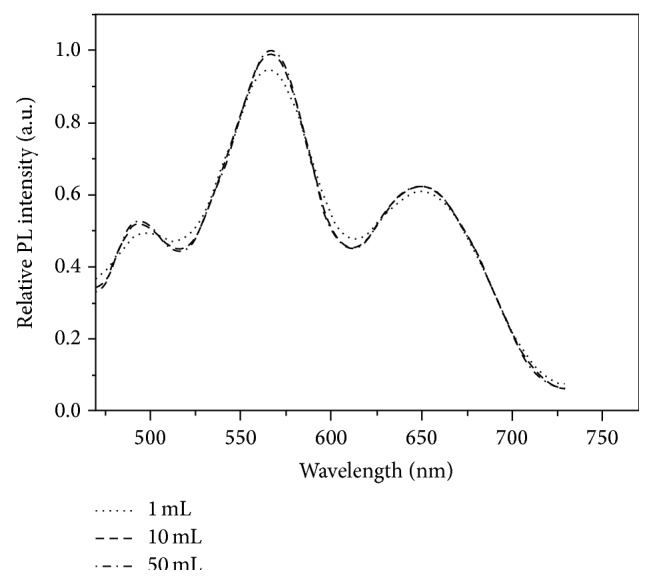
The fluorescence spectra of the hybridization complex separated from above 1 (dotted line), 10 (dashed line), and 50 (dotted and dashed line) mL solutions.

To verify the magnetic separation and enrichment capacity of AFMFEM, the same amounts of 100 *μ*L of HAV Target DNA (50 pM), FITC-Complement DNA, and capture DNA-AFMFEM-1 (15 *μ*L of AFMFEM saturated by capture DNA) were added into different volumes of BB solution (1, 10, and 50 mL) at room temperature. The amount of capture DNA-AFMFEM-1 was kept excess, and the final concentration of HAV Target DNA in the solution was 5, 0.5, and 0.1 pM, respectively. After stirring at room temperature for 2 h, the precipitation of each sample was separated by a magnet and was dispersed in 1 mL PBS buffer solution.  Figure 4 shows the fluorescence spectra of resultant complex. As shown in Figure 4, there are three similar fluorescence peaks in the fluorescence spectra of the products which are fabricated by enrichment and separation of AFMFEM-1-HAV capture DNA in three different samples. The peak at 510 nm is the fluorescence peak of FITC-Target DNA; the peaks at 560 nm and 650 nm are the fluorescence peak of capture DNA-AFMFEM-1, and the fluorescence intensity of three fluorescence peaks at 510 nm is the same, which indicates that the FITC-Target DNA dispersed in 1, 10, mL or 50 mL BB solution can be enriched completely and separated by a magnet same as to the FITC-Target DNA dispersed in 1 mL BB solution. The results imply that 0.1 pM FITC-Target DNA could be detected sensitively. The reported detection limit of other technologies based on nanoparticles is 4 nM for QDs [28], 10 pM for Fe_3_O_4_/Eu:Gd_2_O_3_ [29], 5 pM for cyanide dye-doped silica nanoparticles DNA microarrays [30], and 1 pM for scanometric detection of gold-silver-enhanced nanoparticles in DNA microarrays [31]. In comparison, our analysis method provides a high sensitive nanoimmunoassay platform for enrichment, detection, and separation of trace DNA. With the improvement the performances of magnetic fluorescent encoded nanoparticles, the sensitivity will be further improved, and the nanoimmunoassay technology will be used for detection and analysis of other viruses in the future.

### 3.5. Study of Hybridization Kinetics

**Figure 5:**
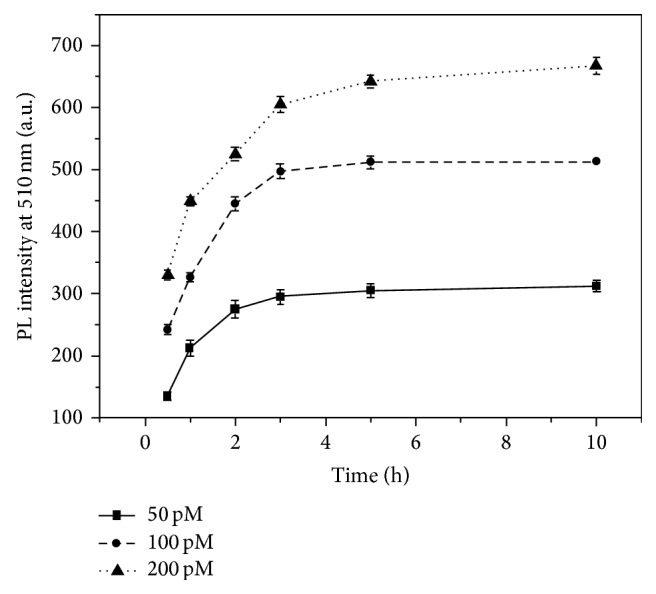
Hybridization kinetics curve with the concentration of target DNA was 50 (■), 100 (∙), and 200 pM (▴), respectively.

For studying the hybridization kinetics of AFMFEM, in this work, the same volume of different concentration of Target DNA was reacted with FITC-Complement DNA and 50 *μ*L capture DNA (50 *μ*L of AFMFEM saturated by capture DNA). The fluorescence spectra were obtained at different hybridization time, as schematically outlined in Figure 5. Although the amount of capture DNA-AFMFEM probe was constant, the reaction time of various concentrations of target DNA hybridized with capture DNA-AFMFEM was different. The hybridization time for obtaining stable composite was short with decrease of the concentration of target DNA, the reaction time of 50 pM Target DNA hybridized with capture DNA-AFMFEM was only 1 h, and the reaction time of 200 pM Target DNA hybridized with capture DNA-AFMFEM was about 3 h.

### 3.6. Detection of Pathogenic DNA Using Fluorescent Sandwich Immunoassay Principle

**Figure 6:**
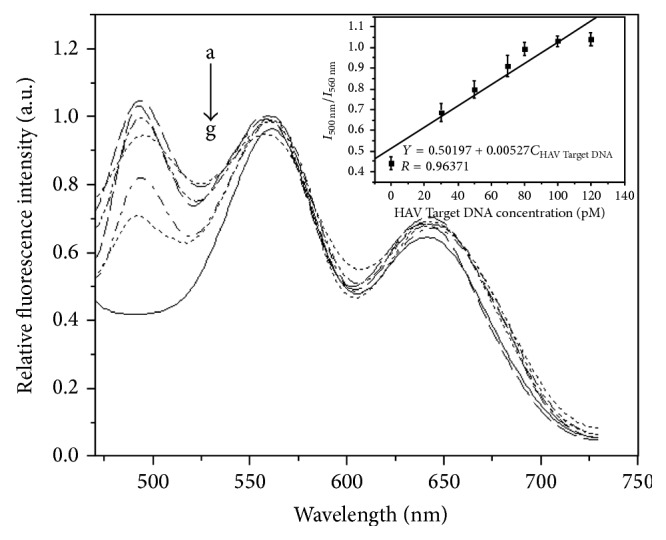
Fluorescence emission spectra of hybridization complex with a series of different concentrations of HAV Target DNA added (a–g: 0 pM, 30 pM, 50 pM, 70 pM, 80 pM, 100 pM, and 120 pM). The inset shows the relationship between relative fluorescent intensity of *I*_500 nm_/*I*_560 nm_ after immunoreaction and the concentration of HAV Target DNA.

**Figure 7:**
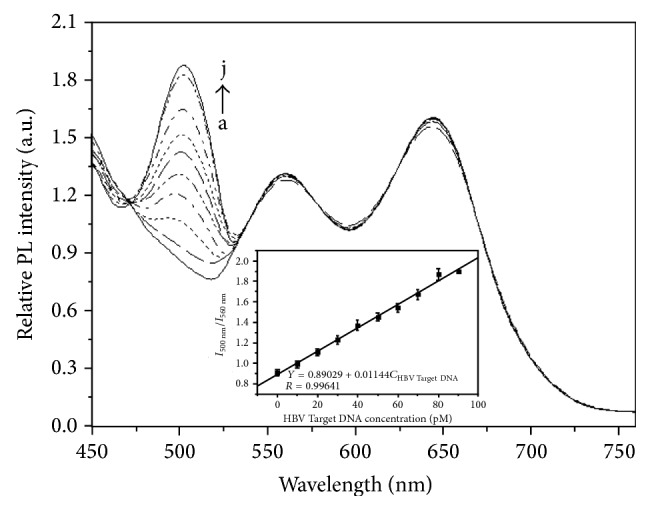
Fluorescence emission spectra of hybridization complex with a series of different concentrations of HBV Target DNA added (a–j: 0 pM, 10 pM, 20 pM, 30 pM, 40 pM, 50 pM, 60 pM, 70 pM, 80 pM, and 90 pM). The inset shows the relationship between relative fluorescent intensity of *I*_500 nm_/*I*_560 nm_ after immunoreaction and the concentration of HBV Target DNA.

In order to detect HAV Target DNA and HBV Target DNA in this work, sandwich immunoassay technology was adopted to analyze and detect unlabeled pathogenic DNA in the solution. The different concentrations of HAV Target DNA in the range of 0–120 pM were added into mixed solution of HAV FITC-Complement DNA and capture DNA-AFMFEM-1 (50 *μ*L of AFMFEM saturated by capture DNA), and the fluorescence spectra of the product were obtained by fluorescence spectrophotometer.  Figure 6 shows the relationship of fluorescent intensity and the amount of HAV Target DNA conjugated on the surface of AFMFEM-1 by electrostatic interaction. The illustration of Figure 6 exhibits the relatively fluorescent intensities (*I*_500 nm_/*I*_560 nm_) versus the concentrations of pathogenic DNA. As we expected, the relationship of the concentration of HAV Target DNA in the linear range from 0 to 100 pM and the relatively fluorescence intensity (*I*_500 nm_/*I*_560 nm_) of the product is proportional linear relationship. The linear regression equation is as follows: *I*_500 nm_/*I*_560 nm_ = 0.50197 + 0.00527*C*_HAV Target DNA_ (pM), and the coefficient of correlation is 0.96371, and the limit of detection for immunoassay is 0.1 pM of HAV Target DNA. Upon further addition of HAV Target DNA, there is no obvious change of fluorescent intensity (*I*_500 nm_/*I*_560 nm_). For the detection of HBV Target DNA, a similar process was used. From Figure 7, it can be seen that the fluorescence intensity at 500 nm also increases gradually with increasing concentration of HBV Target DNA in the range from 0 to 90 pM. The fluorescence intensity of complex emission at 500 nm reaches a plateau when the concentration of HBV Target DNA reaches 90 pM, which indicates the immunointeraction among FITC-Complement DNA, Target DNA, and capture DNA-AFMFEM-2 is complete at this concentration. The linear regression equation is as follows: *I*_500 nm_/*I*_560 nm_ = 0.89029 + 0.01144*C*_HBV Target DNA_ (pM), and the coefficient of correlation is 0.99641, and the limit of detection for immunoassay is 0.12 pM of HBV Target DNA.

### 3.7. Detection of Pathogenic DNA in Human Serum Samples

To assess the application of this nanoimmunoassay system in complex biological systems, the analyses of pathogenic DNA HAV and HBV in human serum samples were carried out. Serum is what remains from whole blood after coagulation; the chemical composition is similar to plasma but does not contain coagulation protein [32]. We performed detection of pathogenic DNA in 10-fold-diluted serum samples under the optimal conditions. The results obtained by standard addition method were listed in Table 1. From the table, we can see that the RSD was lower than 3% and the average recoveries of pathogenic DNA HAV and HBV in the real samples were in the range of 98.7–103.0%, indicating that the accuracy and precision of the proposed method were satisfactory.

## 4. Conclusion

In this work, we have reported a novel strategy for multiplexed immunoassay with magnetic fluorescent encoded composite nanoparticles. The magnetic fluorescent encoded composite nanoparticles, which contain two colors of CdTe QDs and supermagnetic Fe_3_O_4_ nanoparticles for fluorescent encoded and excellent magnetic response property, are a new powerful tool for biological applications. Based on this novel nanomaterial, in this study, we establish a new fluorescent analytical method for multicomponent biological immunoassay by the combination of amino-modified magnetic fluorescent encoded nanoparticles and sandwich immunoassay principle. And this method was successfully applied for the detection and separation of two types of subtrace pathogenic DNA. Compared with the traditional method, the proposed method is time-saving and easy to operate and has high sensitivity (0.1 pM for HAV DNA and 0.12 pM for HBV DNA), and most importantly it can be used for multiplex immunoanalysis and could be applied in many other antibody-antigen systems and virus detection.

## Figures and Tables

**Table 1:** The detection of pathogenic DNA in human serum samples.

Serum samples	Added (pM)	Founded (pM)	Recovery (%)	RSD (% *n* = 3)
1	HAV 1.5 pM	1.48 ± 0.04	98.67	1.06
	HBV 1.5 pM	1.54 ± 0.03	102.67	2.18

2	HAV 2.0 pM	2.06 ± 0.02	103.00	1.83
	HBV 2.0 pM	2.03 ± 0.05	101.50	0.92

3	HAV 3.0 pM	3.08 ± 0.06	102.67	1.10
	HBV 3.0 pM	3.09 ± 0.05	103.00	1.22
